# Gene expression profiles of prostate cancer reveal involvement of multiple molecular pathways in the metastatic process

**DOI:** 10.1186/1471-2407-7-64

**Published:** 2007-04-12

**Authors:** Uma R Chandran, Changqing Ma, Rajiv Dhir, Michelle Bisceglia, Maureen Lyons-Weiler, Wenjing Liang, George Michalopoulos, Michael Becich, Federico A Monzon

**Affiliations:** 1 Departmental of Biomedical Informatics, University of Pittsburgh, Parkvale Building M-183, 200 Meyran Ave, Pittsburgh, PA 15260, USA; 2 Department of Pathology, University of Pittsburgh, S-417 BST, 200 Lothrop Street, Pittsburgh, PA 15261, USA

## Abstract

**Background:**

Prostate cancer is characterized by heterogeneity in the clinical course that often does not correlate with morphologic features of the tumor. Metastasis reflects the most adverse outcome of prostate cancer, and to date there are no reliable morphologic features or serum biomarkers that can reliably predict which patients are at higher risk of developing metastatic disease. Understanding the differences in the biology of metastatic and organ confined primary tumors is essential for developing new prognostic markers and therapeutic targets.

**Methods:**

Using Affymetrix oligonucleotide arrays, we analyzed gene expression profiles of 24 androgen-ablation resistant metastatic samples obtained from 4 patients and a previously published dataset of 64 primary prostate tumor samples. Differential gene expression was analyzed after removing potentially uninformative stromal genes, addressing the differences in cellular content between primary and metastatic tumors.

**Results:**

The metastatic samples are highly heterogenous in expression; however, differential expression analysis shows that 415 genes are upregulated and 364 genes are downregulated at least 2 fold in every patient with metastasis. The expression profile of metastatic samples reveals changes in expression of a unique set of genes representing both the androgen ablation related pathways and other metastasis related gene networks such as cell adhesion, bone remodelling and cell cycle. The differentially expressed genes include metabolic enzymes, transcription factors such as Forkhead Box M1 (*FoxM1*) and cell adhesion molecules such as Osteopontin (*SPP1*).

**Conclusion:**

We hypothesize that these genes have a role in the biology of metastatic disease and that they represent potential therapeutic targets for prostate cancer.

## Background

Prostate cancer is the most common cancer in men resulting in over 232,090 new cases and 30,350 deaths annually [1]. For prostate cancer patients, metastatic disease reflects the most adverse clinical outcome. Osseous involvement with severe bone pain and spinal cord complications occur commonly in patients with metastatic disease [2]. However there is considerable heterogeneity in outcome after primary diagnosis and currently there are no morphologic or circulating biomarkers that can accurately predict the development of metastatic disease.

Metastatic prostate cancer represents the tumor's ability to escape from the primary organ and eventually colonize a distant site. Disruption of a complex set of biological processes must occur in order for tumor cells to leave the prostate and establish themselves in a different environment. Their altered interaction with the prostate microenvironment, including the stroma and extracellular matrix, their ability to migrate into the vasculature and establish themselves in secondary organs with recruitment of vascular supply represent disruption of normal cellular processes [3]. Understanding the molecular events involved in the development of metastatic prostate cancer has the potential to identify biological determinants that can aid in prognosis and development of more effective therapies.

Using gene expression microarrays, a number of studies have characterized expression profiles of prostate cancer, normal tissue and metastatic cancers. In some cases, correlations between tumor expression signatures, clinical parameters and outcome have been identified [4-11]. Unique profiles have been reported for untreated and short-term androgen ablation treated organ-confined disease and for metastatic disease, with a subset of genes differentiating metastatic androgen ablation resistant prostate cancer (AARPC) from androgen dependent metastatic cancers [10,12-14]. In general, metastatic prostate cancer is characterized by changes in expression of genes involved in signal transduction, cell cycle, cell adhesion, migration and mitosis. In addition to these genes, AARPCs exhibit changes in expression of the androgen receptor and enzymes involved in the sterol biosynthesis pathway [12].

Some of the genes previously reported as highly downregulated in prostate tumors may reflect the differences in cellular content of metastatic and organ-confined tissues rather than intrinsic differences in biology. In contrast with organ-confined prostate tumors which are composed of a mixture of glandular epithelial, smooth muscle and other stromal cells, metastatic tissue samples are almost exclusively epithelial, with minimal supporting stroma and absence of smooth muscle. In this study, we characterize gene expression in androgen ablation resistant metastatic tumors after removing potentially uninformative stromal genes. The deleted stromal genes consist of those reported in a recent report characterizing the gene expression patterns in the prostate stroma, tumor and normal epithelium [15]. Our results provide novel insights into the biology of metastasis.

## Methods

### Tumor sample procurement

All tissue samples were acquired from the Health Sciences Tissue Bank of the University of Pittsburgh Medical Center under stringent Institutional Review Board guidelines with appropriate informed consent. The 18 donor and 64 primary prostate tumor samples have been described previously [7]. Specimens were received directly from the operating room. Samples (>500 mg) were excised and snap frozen in liquid nitrogen within 30 min of excision and stored at -80°C until extraction of RNA. Metastatic tumor samples were obtained from a warm autopsy program and processed similarly to primary tumors. An H&E stained frozen section of each sample was evaluated by a pathologist, to determine epithelial and stromal content and verify the presence of tumor in the sample. Dissection of the frozen tissue block was performed with the guidance of a marked H & E slide to minimize the presence of host tissue in the metastatic samples. All samples used in the study contained >80% tumor. Metastatic tumor samples were minced and divided into two equal portions to be extracted with the sample protocol used for each set of primary tumors.

### Clinical profile of cases

The clinical characteristics of the 64 primary tumor samples used in the Affymetrix portion of our study have been previously described [6,7]. These cases have a mean follow-up time of 3 years. The metastatic samples consisted of 24 tissues derived from 4 patients (Table 1). All patients with metastatic disease had received androgen ablation therapy and had shown progression of disease while on androgen ablation. The clinical characteristics for the additional 10 primary prostate tumor cases used in the CodeLink study are shown in Table 1.

### RNA extraction

RNA purification for the 64 primary samples has been previously described [6]. The set of metastatic samples analyzed with the Affymetrix platform was extracted with the same methodology. The set of metastatic samples and primary tumors analyzed with the CodeLink platform were extracted using the RNeasy kit (Qiagen, San Diego, CA). For the metastatic samples, one sample did not have enough for extraction with the Qiagen method, only 23 metastatic samples are included in the CodeLink assays. The concentration of each total RNA sample was measured with a Nanodrop ND-1000 spectrophotometer (Nanodrop Technologies,Wilmington, DE). RNA integrity was determined by capillary electrophoresis using an Agilent 2100 Bioanalyzer (Agilent, Willmington, DE).

### cRNA preparation and gene expression assays

cRNA was prepared and hybridized to Affymetrix GeneChip HGU95av2, HGU95b and HGU95c arrays (Affymetrix, Santa Clara, CA) as previously described [6]. For gene expression profiling with the CodeLink Gene Expression System (GE Healthcare, Piscataway, NJ), biotin-labeled cRNA was prepared as previously described [16]. Ten micrograms of biotin-labeled cRNA product from each sample were then fragmented with RNA fragmentation buffer at 94°C for 20 minutes. Hybridization mix was prepared according to the manufacturer's instructions and the final volume was adjusted to 260ul using nuclease-free water. The hybridization mix was heat denatured at 90°C for 5 minutes, cooled on ice and then applied to Human Uniset 20 K arrays (GE Healthcare, Piscataway, NJ). Arrays were incubated at 37°C for 18 h with shaking at 60 rpm in an Innova hybridization oven (New Brunswick, Edison, PA).

After hybridization, arrays were placed in a pre-heated (46°C) chamber filled with 0.75 × TNT (0.75 M Tris-HCL, *p*H 7.6, 3.75 M NaCl, Tween-20, and milli-Q water) and incubated at 46°C for 1 hour. Arrays were then stained with Streptavidin-Alexa Fluor 647 (Molecular Probes, Grand Island, NY) for 30 minutes at room temperature. Upon the completion of staining, the arrays were washed three sequential times in fresh 1 × TNT (1 M Tris-HCL, *p*H 7.6, 5 M NaCl, Tween-20, and milli-Q water) and then washed two final times in fresh solutions of 0.05% Tween-20 and 0.1 × SSC with gentle agitation. All arrays were dried by centrifugation at 2000 rpm for 3 minutes.

Affymetrix arrays were scanned in an Affymetrix GCS3000 Scanner (Affymetrix, Santa Clara, CA). CodeLink arrays were scanned with the GenePix 4000B scanner using GenePix Pro 4.1 software (Molecular Devices, Sunnyvale, CA).

### Gene expression data analysis

The raw scanned array images from the Affymetrix GeneChip U95 arrays were processed using GCOS 1.1 software using the MAS5 algorithm (Affymetrix Corporation, Santa Clara, Ca) to generate probe cel intensity (*.cel) files. Data normalization to remove variation in overall chip intensities was performed by global scaling to a chip mean target intensity of 200 (MAS 5.0). Data for U95Av2, B and C arrays were combined for further analyses.

To identify differentially regulated genes in both datasets, these were analyzed with the Significance Analysis of Microarrays software (SAM v 1.2) [17]. Prior to analysis, genes that showed low variation across all samples were removed by using the filtering option in the Avadis 3.3 Pride Software (Strand Life Sciences, Bangalore, India) data analysis tool. To avoid false results due to difference in the tissue composition of metastatic and primary tumors, genes identified as being highly expressed in the prostatic stroma as per Stuart *et al *[15] were also removed. In all 1506 stromal genes and 7678 invariant genes were removed from the Affymetrix dataset. SAM generated gene lists with the lowest false discovery rates (FDR) were further analyzed for gene ontology (GO) and pathway annotations using NIH's DAVID annotation tool [18].

For CodeLink arrays, image files were analyzed with the CodeLink Expression Analysis Software version 4.1 (GE HealthCare) with use of the normalized intensity values in downstream analysis. For cross-platform comparison, Affymetrix probe sets and Codelink identifiers were mapped to Unigene ids using the DAVID annotation tool (see above). Expression data from both platforms was compared using z-transformation. Hierarchical clustering was performed using Eisen's Cluster and Treeview [19]. Data from Affymetrix experiments has been submitted to NCBI's Gene Expression Omnibus (GEO) as series GSE6919, with the following accession numbers GSE6604 (normal donor prostate), GSE6605 (metastatic prostate tumors), GSE6606 (primary prostate tumors) and GSE6608 (normal prostate tissue adjacent to tumor). Data from the CodeLink platform have been submitted to GEO with the accession number GSE6752 (primary and metastatic prostate tumors).

### Quantitative real-time PCR

Differential expression of ten genes in primary and metastatic prostate cancer samples was verified with quantitative real-time PCR (QPCR) with the ABI PRISM^® ^7000 sequence detection system (Applied Biosystems, Foster City, CA). Three selected RNA samples from each patient were pooled together (except for patient FB666 n = 1) and therefore four RNA samples, each representing one patient, were tested. RNA samples were first heat-denatured at 70°C for 10 minutes in an Eppendorf master cycler (Eppendorf, Westbury, NY) and then chilled immediately on ice. cDNAs were reversely transcribed from one microgram of RNA using the M-MLV Reverse Transcriptase kit (Invitrogen, Carlsbad, CA), as recommended by the manufacturer. QPCR was performed based on the manufacturer's instructions with TaqMan Gene Expression Assays (Applied Biosystems) for the following genes: EGR3, SYNPO2, ANGPT2, SPP1, FOXM1, ADM, RDX, TGFBRAP1, MAK and EGR1(assay IDs: Hs00231780_m1, Hs00326493_m1, Hs01048047_mH, Hs00959010_m1, Hs00153543_m1, Hs00969450_g1, Hs00988414_g1; Hs01093285_m1; Hs01048300_m1, Hs00152928_m1). When multiple TaqMan assays for one gene were available, the assay that interrogated the sequence closest to the target sequence in the Affymetrix arrays was chosen. PCR cycles were performed according to the assay instructions in an ABI PRISM^® ^7000 Sequence Detection System (Applied Biosystems). Relative quantification of the expression level of each transcript in each sample was calculated using the Delta-Delta CT method in the ABI PRISM 7000 Sequence Detection System Software (Applied Biosystems) [20]. Human reference RNA from Stratagene (Stratagene Corp., La Jolla, CA) was used as the calibrator (untreated control) and human glucuronidase beta (GUSB) gene was used as the endogenous reference gene (Forward primer: GGA ATT TTG CCG ATT TCA TGA; Reverse primer: CCG AGT GAA GAT CCC CTT TTT; Probe: 6FAM-AAC AGT CAC CGA CGA GAG TGC TGG G-TAMRA).

## Results

### Differential gene expression in metastatic prostate cancer and the role of stromal content in defining true downregulated genes

Differential expression analysis of the metastatic and primary tumor samples shows that a large number of the most highly downregulated genes such as *TAGLN*, *ACTG2*, *TPM1*, *MYH111 *and *DES *have been previously identified as expressed mostly in the prostatic stromal cells [15]. Since only the epithelial component of prostate cancer is present in metastatic tumors, this result most likely reflects the lack of stroma in metastases, and not a true down-regulation of these genes in the metastatic epithelial cells. Therefore, based on a recent report characterizing cell type specific gene expression in the prostate [15], we removed the set of genes expressed mainly by the stromal cells of the primary tumors. In all 1506 transcripts associated with a stromal signature were deleted prior to further analysis. Since the stromal genes were characterized using the U95Av2 chip and our analysis includes u95Av2, B and C chips, only stromal genes represented by probe sets on U95Av2 were removed in this modified analysis. SAM analysis shows that 1277 genes are up and 977 genes are downregulated at least 2 fold at the lowest FDR (0.01), in metastatic prostate samples (see Additional file 1). A list of the top 50 up and top 50 down regulated genes at the lowest fdr, after removing ESTs and uncharacterized clones is shown in Table 2. This list includes signal transducers, cell cycle regulators, metabolic enzymes and cell adhesion molecules. Some of the most upregulated genes in our list are *EIF1AX*, *AR*, *HSPD*1 and *HSPCA*, *K-ALPHA1*, *MLL5*, *UGT2B15*, and some of the most downregulated genes include *WNT5B5, ANXA11, FOS *and *SFRP1*.

### Metastatic samples are heterogenous in gene expression

**Figure 1:**
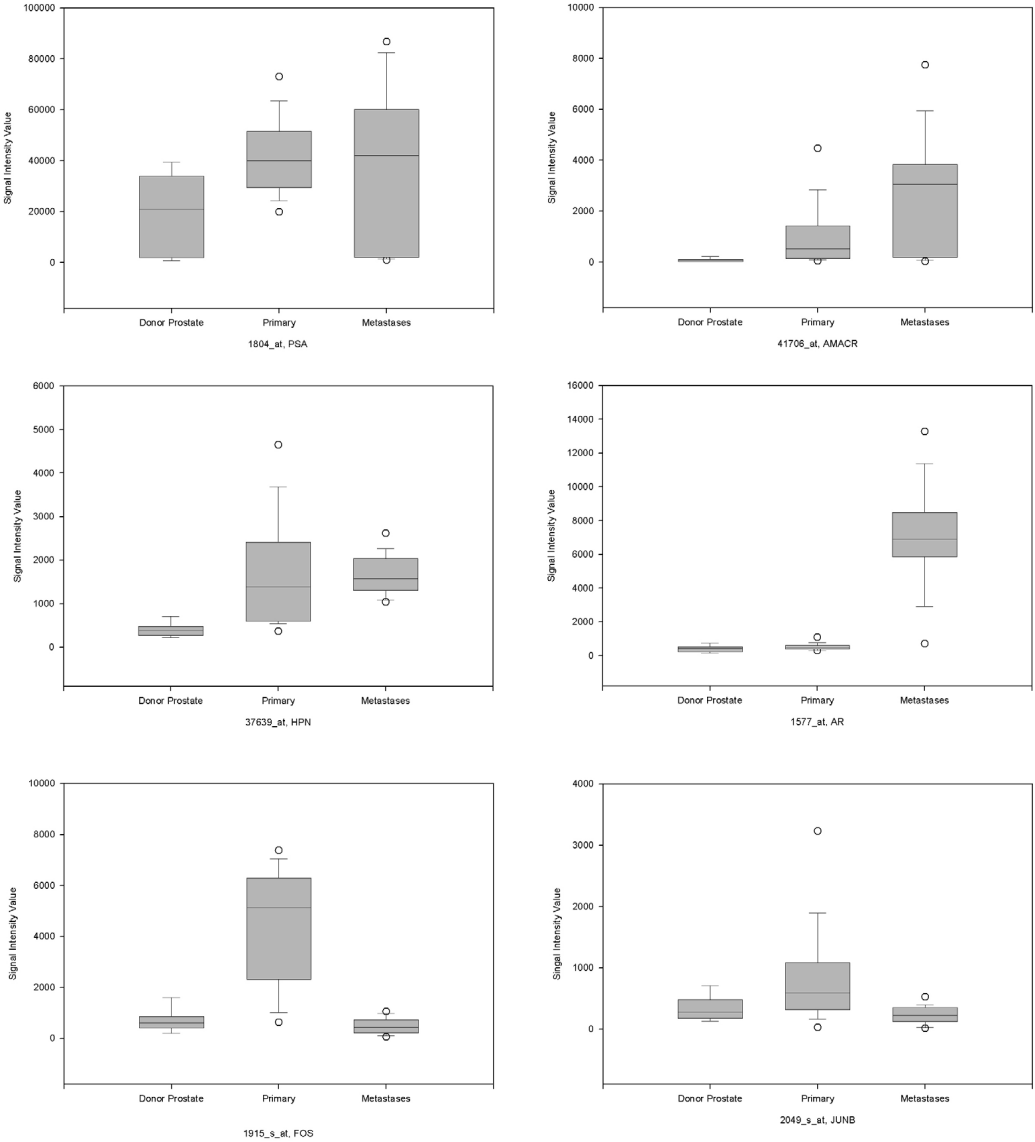
Box plots of gene expression values for selected genes in donor prostate samples, primary prostate cancer and metastatic prostate samples.

Using immunohistochemistry (IHC) Shah *et al*. have shown that metastatic samples are highly heterogenous in expression of prostate specific markers leading to the hypothesis that at the molecular level, metastatic prostate cancer may represent multiple diseases even within the same patient [21]. We examined the expression of several transcripts markers including some studied by Shah *et al*. and confirmed the heterogeneity of expression levels in metastatic prostate cancer tissues. Expression values in donor samples, primary and metastatic samples were compared. Prostate specific antigen (*PSA*/*KLK3*) remains high in some metastatic samples and is low or absent in others, even within the same patient (Figure 1). Interestingly, *AMACR*, another biomarker for prostate cancer [22] expresses a heterogenous expression pattern similar to *PSA*. *HPN*, which is overexpressed in primary cancer maintains high expression in the metastatic samples in our study. *AR*, while overexpressed in 23 out of the 24 metastatic samples, shows highly variable expression values in individual samples. The proto-oncogenes *FOS *and *JUNB*, which are both overexpressed in primary tumors, are consistently downregulated in all metastatic samples.

### Genes regulated in all metastatic cases

**Figure 2:**
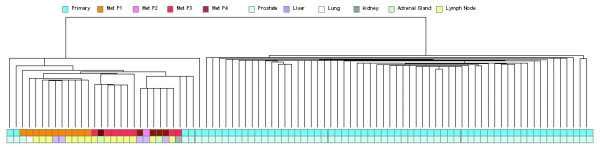
**Hierarchical clustering of primary and metastatic prostate cancer samples**. The 24 metastatic (Mets P1, Mets P2, Mets P3 and Mets P4) and 64 primary tumor samples were clustered. The top row of color coded boxes represents metastatic or primary samples; the bottom row represents the organ from which the sample was obtained.

Hierarchical clustering analysis reveals that gene expression in metastatic samples is more variable between patients than between different metastatic sites from each patient (Figure 2). Although the 24 metastatic samples represent tissues from 6 metastatic sites (Table 1), no organ specific clusters were detected (Figure 2) whereas samples from the same patient tend to cluster together. Statistical comparison of organ-specific expression profiles was not attempted due to unequal distribution of samples from different metastatic sites.

In order to identify probe sets that are similarly regulated in every patient, and therefore likely to represent a specific metastatic profile, the SAM differentially expressed gene list at a FDR of 2% was further filtered. For each gene on this list, a patient specific median expression value was calculated from the multiple samples from each patient. Patient P4 had only sample and this sample's signal value was considered the median value. The median values were then compared to the median value of the primary samples and those probe sets whose median value showed equal or more than a 2 fold change in every patient were considered part of the metastatic prostate cancer signature. Under this criteria 415 transcripts are upregulated and 364 are downregulated in all patients with metastasis (see Additional file 2). A truncated gene list consisting of genes regulated at least 3 fold in all patients is shown in Table 3. Upregulation of *AR *in all samples from metastatic cancer patients represents a known "androgen resistant" or AARPC (androgen ablation resistant prostate cancer) phenotype [12]. The transcripts identified as differentially expressed in our study exhibit similarities with a previous study of AARPC tumors [10,12]. Cytokeratins 5 and 15 (*KRT5*/*KRT15*), markers of basal cells in prostate glands, show uniform downregulation in all metastatic tumors, confirming the absence of basal epithelial cells.

### Biological annotation of differentially expressed genes in metastatic prostate cancer

The list of differentially expressed transcripts at least 2 fold in all patients was further analyzed for biological themes and gene ontology (GO) using the NIH's DAVID annotation tool. This analysis revealed that metastatic prostate cancer exhibits altered regulation of amino acid, carbohydrate and nucleotide metabolism consistent with the proliferative capacity and altered energy needs of metastatic tumors (data not shown). In the context of prostate cancer biology, genes involved in cell-adhesion, bone remodeling, cell-cycle and transcription are of particular interest (Table 4). Disruption of cell adhesion and altered interaction with the extracellular matrix is a hallmark of metastatic tumors [3]. In agreement with this, the secreted phosphoprotein and cell adhesion molecule osteopontin (SPP1) is one of the most highly upregulated transcripts in our metastatic samples. Elevated expression of SPP1 has been correlated with poor prognosis in prostate tumors and other cancers and it has often been implicated in metastasis to bone and other organs [2,23-28]. In all 29 probe sets representing cell adhesion genes are altered in all metastatic samples. This gene list includes *FN1*, *ITGB8*, *THBS*2, *HNT *and *CDH10*. Genes involved in bone remodeling such as *BMP4 *and *ANKH *are also altered in expression, although none of the samples in our study are bone metastatic samples suggesting that these proteins may also be involved in cancer metastasis to other organs.

Disruption of the cell cycle is highlighted by the presence of a large number of cell-cycle related transcripts in the list of differentially expresed genes in all metastatic samples. The list contains 37 cell cycle genes, and includes *SEP4*, *SEP7*, *PTN *and *VEGF*. Similarly, a large number of transcription factors (67) including *AR*, *SRY*, *FOS *and *EGR3*, are differentially expressed. Two members of the winged-helix family of transcription factors, *FoxP1 *and *FoxM1*, show upregulation in the metastatic samples. Interestingly, *FoxM1b *has been shown to promote progression of prostate carcinomas in an experimental model [29].

The MAP kinase signaling pathway was also identified as being important in the metastatic process, with 26 probe sets involved in this pathway being differentially expressed in all metastatic samples. The regulated genes include *DUSP1*, *DUSP2*, *DUSP8*, *MAP3K8*, *MAP4K4*, *FGF13*, *FGFR2 *and *FOS*. Involvement of MAP kinase in androgen receptor signaling has been previously described [30].

### Validation of differentially expressed transcripts with an independent set of primary tumors and different gene expression platforms confirms gene expression profiles of metastatic prostate cancer

**Figure 3:**
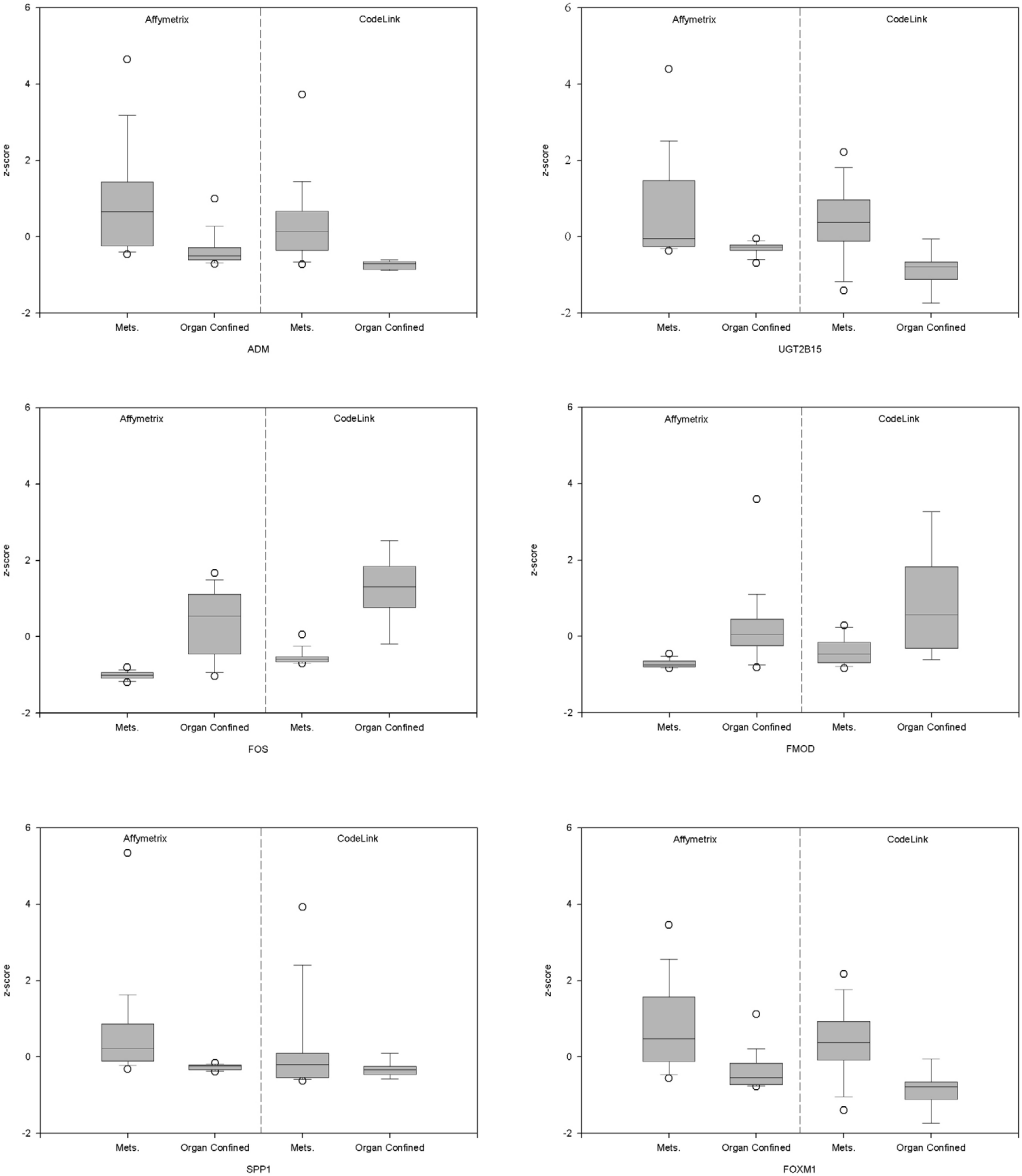
**Comparison of Z-transformed expression values between the Affymetrix and Codelink platforms**. Gene expression data from Affymetrix and CodeLink experiments was Z-transformed to allow comparison. Data for selected differentially expression genes is shown.

Gene expression analysis with the CodeLink Uniset 20 K microarray was carried out for 23 of the metastatic samples and compared to an independent set of 10 primary tumors. Similar to the Affymetrix analysis (see above), hierarchical clustering of the CodeLink data set reveals heterogeneity in expression and no organ-specific clustering (data not shown). Comparison of results with the Affymetrix based dataset, based on genes with common Unigene ids on both platforms, show a similar pattern of differentially expressed genes. Of the top 1000 up and down regulated transcripts from each platform, approximately 70% share common unigene ids and of these 22% of the genes are identified as regulated by both platforms (see Additional file 3). This level of correlation is significant, given the well-documented difficulties in cross-platform comparisons of expression data [31,32]. Examples of z-transformed expression values for selected genes in both platforms are shown in Figure 3.

**Figure 4:**
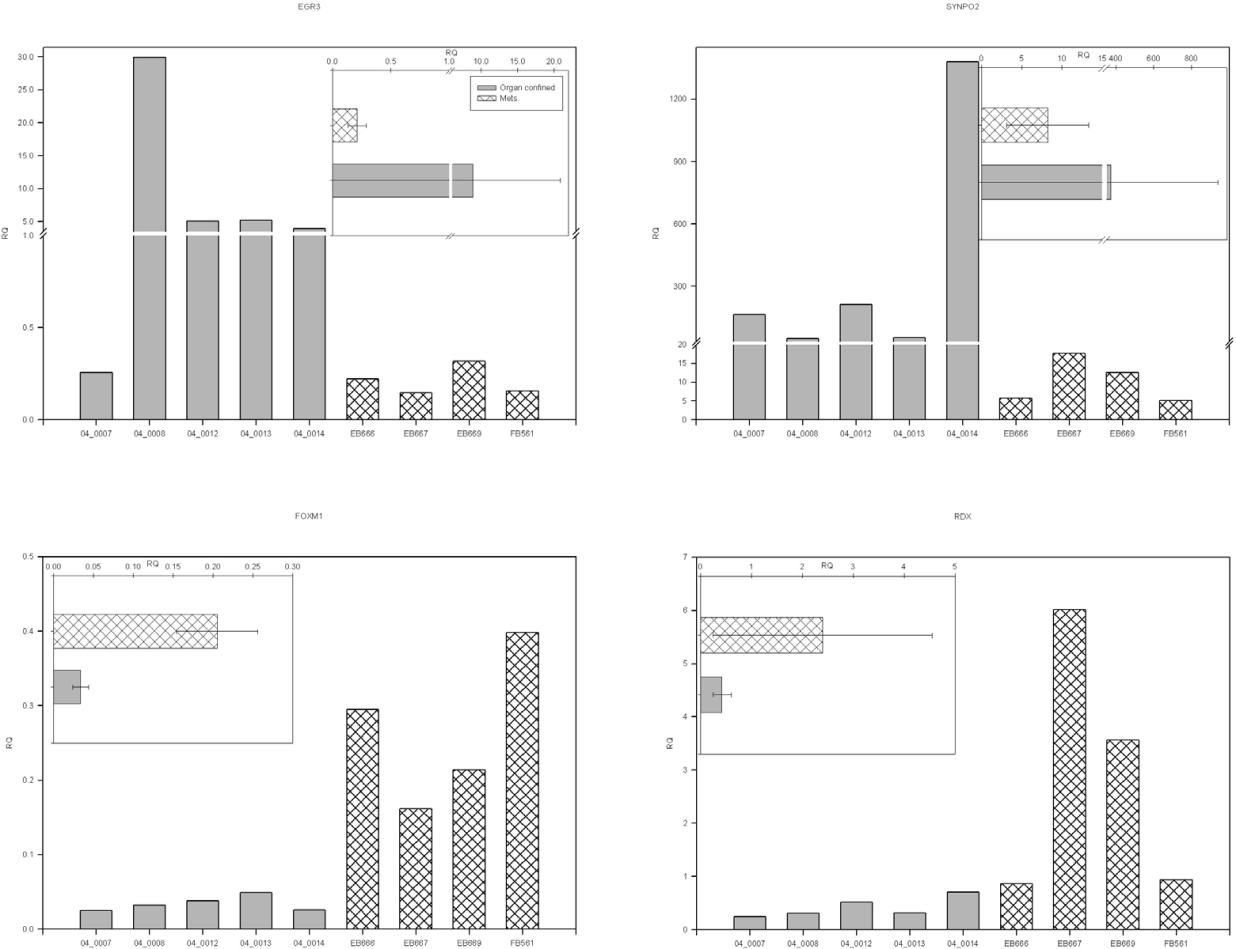
**Validation of differentially expressed genes with quantitative real time PCR**. QPCR was performed on RNA from samples used in the CodeLink analysis. Three selected metastatic RNA samples from each patient were pooled together (except for patient FB666 n = 1) and therefore four RNA samples, each representing one metastatic patient and 5 primary tumors were tested. The insert shows average expression values for metastatic and primary tumors.

Additionally, real-time quantitative PCR (QPCR) assays were performed for a selected set of genes in pooled samples for each patient with metastatic disease and 5 of the primary tumors from the CodeLink set. The transcripts for this analysis were chosen to represent diverse biological processes and were chosen from the differentially expressed genes identified as up/down-regulated in the Affymetrix/Codelink data comparison. As shown in Figure 4, qPCR assays confirmed the results from the microarray platforms. *SYNPO2 *and *EGR3*, which are downregulated and *RDX *and *FOXM1*, which are upregulated in the microarray analysis exhibit a very similar expression pattern in the qPCR analysis. Interestingly, *FoxM1 *is consistently upregulated in metastases, while RDX was upregulated in only two of the four patients with metastatic disease, confirming the heterogeneity of metastatic prostate cancer.

## Discussion

Despite extensive research, the molecular mechanisms of metastatic prostate cancer and androgen resistance development are still poorly understood. Our study shows that a number of biological processes including cell adhesion, cell cycle and transcription regulation are altered in metastatic disease when compared to primary tumors, and point to specific transcripts that participate in the metastatic process.

Previous investigators have reported differences in gene expression profiles of metastatic and primary prostate cancer [10,12,14,21]. Our results show partial overlap with these previous characterizations of metastatic disease. Some genes that are in concordance with these studies include transcription factors such as *FOXM1*, and c-*FOS.1*. Differences in patient demographics, pathology and treatment, non-standard tissue handling, experimental and statistical methods may all contribute to differences in gene lists. Differences with other published gene lists might also reflect the fact that in our study, only samples from patients with androgen-insensitive prostate cancer were used. Additionally, in our experimental design we have incorporated features that increase the significance of our findings and increase the likelihood that the genes identified truly reflect the biology of metastatic prostate cancer. First, by subtracting transcripts previously identified as being expressed by the prostatic stroma, we have incorporated previous knowledge about the expression profiles of different components of prostate tumors in order to focus on those transcripts intrinsic to metastatic cells. This takes into account the fact that metastatic tumors do not contain all the tissue elements present in organ-confined tumors. The major benefit of this strategy is to better define the genes that are down-regulated in metastatic tumor cells. Second, by analyzing multiple tumor samples from each patient, we have addressed the fact that metastatic prostate cancer shows significant heterogeneity, even within the same patient [21]. This is corroborated by our results. and we address this issue by focusing our analysis on the transcripts that show significant differential expression in all metastatic sites within and between patients.

Multiple biological processes appear to be altered in metastatic prostate cancer. One common theme that has emerged from studies of metastatic disease is the central role of the androgen receptor in the development of androgen resistant disease. Several mechanisms including amplification of the *AR *gene, upregulation of mRNA expression to allow binding by low levels of androgens, mutations in the ligand binding domain (LBD) that allow the receptor to be activated by antagonists, and alteration in the normal *AR *signaling pathway, have been proposed to explain the ability of prostate cancer to recur in the presence of androgen ablation therapy [33,34]. Consistent with previous observations, *AR *is up-regulated in all metastatic samples in our study. Similarly, gene expression changes of the MAP kinase pathway in metastasis may be related to the development of the AARPC phenotype. The gene list from our analysis shares some similarities with mouse xenograft prostate cancers models (CWR22) of androgen independence. *MSMB*, *CCND1*, *EFNA3*, *FKBP *and *ADM*, *HGF *are similarly regulated in mouse models and in our study [35-37].

Changes in the expression level of several additional transcripts may reveal clues about the mechanism of metastasis and androgen resistance. We find upregulation of the enzyme *UGT2B15 *in all metastatic patients. Upregulation of *UGT2B15 *in androgen independent prostate cancer has been reported previously [14]. This increase appears paradoxical, since *UGT2B15 *is involved in hormone inactivation. However, as suggested by Stanbrough *et al *[14], upregulation of multiple genes related with androgen metabolism might reflect that metastatic tumor cells have an increased capacity to convert weak androgens into testosterone or DHT. However, in contrast to their findings, transcripts for *AKR1C3*, *SRD5A1*, *HSD3B2*, *AKR1C2*, *AKR1C1 *are not consistently upregulated in the metastatic samples in our study. Interestingly, when reviewing individual values for each sample, some metastases indeed show higher levels for some of these transcripts, which might reflect the heterogeneity of metastatic prostate cancer phenotypes. Another possible explanation for this discrepancy is that the metastatic samples used by Stanbrough *et al*. are all from bone metastases and this type of sample is not represented in our study. Clearly, further investigation into the role of these pathway genes in the development of androgen resistance by metastatic samples is needed.

Several genes involved in cell-cell interaction and cell adhesion appear to be up-regulated in these tumors. *SPP1 *(osteopontin), a secreted, integrin-binding glycoprotein with adhesive properties, has been shown to be correlated with metastasis to the bone and with poor prognosis in various cancers and is highly upregulated in all the metastatic samples in our study. Elevated plasma osteopontin levels have also been correlated with lower survival and bone metastasis in hormone resistant prostate cancer [25]. Interestingly, Stanborough and collaborators also identified *SPP1 *as upregulated in their metastatic samples, however, their interpretation was that this increase was part of the bone response to the metastases. Our study confirms that upregulation of *SPP1 *is a feature intrinsic to androgen-resistant metastatic prostate cancer, independent of the site of metastasis. It has been postulated that metastasis to specific target organs may require not only expression of *SPP1 *but an additional set of signaling molecules that promote metastasis to the specific organ. *SPP1 *when expressed with *IL11 *has been shown to promote metastasis of breast cancer cells to the bone [38] but not to the adrenal medulla. Further detailed studies are required to address the specific role of *SPP1 *and other co-expressed genes in prostate cancer metastasis and whether *SPP1 *represents a potential therapeutic target for androgen-resistant disease. Interestingly, the gene expression profile termed as "bone module" and postulated as a hallmark of tumor metastasis to bone [39] is not dysregulated in our study, most likely reflecting the fact that we did not assay bone metastatic samples. It is also possible, that the role that *SPP1 *plays in metastasis to bone and/or other organs may involve distinct mechanisms [38].

Metastatic tumors have been described as undergoing an epithelial to mesenchymal transition with loss of the differentiated phenotype. Downregulation of transcription factors such as *JUN *has been observed in advanced stages of other cancers and its loss of activity has been postulated to be involved in this transition [40]. In our study, both *FOS *and *JUNB*, which are upregulated in primary tumors compared to normal prostate tissue are highly downregulated in the metastatic samples. *FBN*, also representative of the EMT transition [38] is overexpressed in our metastatic samples. Our analysis has also identified a number of additional genes, such as *KLK11*,*STC1 *and *S100A8 *that are uniformly regulated in all metastatic patients. The role of *S100A8 *in prostate cancer has been studied with evidence suggesting that it is elevated in prostate cancer and may be involved in MAP kinase and *NFK-B *signalling [41,42]. *STC1*, involved in calcium homeostasis, has been reported to have osteoblastic and angiogenic modulator properties with altered expression in some cancers [43-45]. The serine protease *KLK11 *appears to be regulated in prostate cancer with negative correlation between aggressiveness and expression [46].

A recent study observed overexpression of 62 genes due to surgical manipulation related ischemia of the prostate [47]. In our study, 12 out of the 62-gene ischemia profile are downregulated in all metastatic samples. This gene list includes *DUSP1*, *BTG2*, *IER2*, *PTGS2*, *NR4A1*, *AMD1*, *C20orf35*, *KLF4*, *RAB4A*, *KLF6*, *CTGF *and *GOLPH2*. In our data set, these genes represent only 0.01% of the total number of genes differentially regulated in all metastatic samples. Since our metastatic samples all originate from autopsy studies, it is likely that they had been exposed to longer ischemia than the organ confined samples obtained from surgical specimens. Thus, if the differences we observed were related to the ischemia, we would have expected an increase in the expression of these genes, and not the observed downregulation. Therefore, it is unlikely that surgical manipulation can explain the differential gene expression between metastatic and primary tumors.

## Conclusion

In summary, our results support the roles for specific cell adhesion, androgen metabolism and transcription factor genes in the development of androgen-independent metastatic prostate cancer. Furthermore, the differentially expressed transcripts in metastatic tumors that we report have been validated with two independent sets of primary tumors, two gene expression microarray platforms, and selected genes were further validated by qRT-PCR. Our results corroborate the notion that metastatic prostate cancer is quite heterogeneous within a single patient. Despite this heterogeneity our experimental design allowed us to identify common expression profiles for androgen-independent metastatic prostate cancer.

## Abbreviations

Significance Analysis of Microarrays (SAM); false discovery rate (FDR), androgen ablation resistant prostate cancer (AARPC)

## Competing interests

The author(s) declare that they have no competing interests.

## Authors' contributions

URC was involved in data analysis, results interpretation and manuscript preparation. RD was involved in the histologic evaluation of samples. CM performed the QPCR experiments and was involved with data analysis and results interpretation. MLW and WJL performed the Affymetrix and CodeLink experiments. GM and MB participated in conceptualization and study design and manuscript review. FM was responsible for general oversight of the study, providing technical direction, guidance for the analysis team, and participated in manuscript preparation. All authors have read and approved the final manuscript.

## Pre-publication history

The pre-publication history for this paper can be accessed here:



## Supplementary Material

Additional file 1 **Differentially expressed genes between metastatic and primary prostate tumors**. Results of SAM analysis of the 24 metastatic and 64 primary tumors. The Affymetrix probe set id, gene names and assignment of biological process for each gene is shown. Click here for file

Additional file 2 **Comparison of median expression values of all samples from each metastatic patient with primary tumor expression values**. After SAM analysis of the 25 metastatic and 64 primary tumors, genes whose median expression values differ at two in each metastatic patient compared to the median value of primary tumor samples were selected. Click here for file

Additional file 3 Genes identified as regulated in metastatic prostate cancer from both Codelink and Affymetrix platforms Click here for file

## Figures and Tables

**Table 1:** Clinical variables for primary and metastatic prostate cancer samples used in this study

**Prostate Cancer Tissue Samples**	**No. of Samples**	**Number of Patients**	**Microarray Platform**	**Clinical Information**	
	64	64	Affymetrix	Please see reference [7]	
	
				**Gleason Score**	**No. of Cases**
				
				7	10
				
Primary Tumors	10	10	CodeLink	**Pathological Stage**	**No. of Cases**
				
				2B	6
				3A	3
				3B	1

				**Patient ID**	**No. of Samples**
				
				FB6561	11
				FB666	1
				FB667	8
				FB669	5
				
				**Metastatic Sites**	**No. of Samples**
				
Metastatic	24	4	Affymetrix	Liver	5
				Para Aortic Lymph Node	3
				Para Tracheal Lymph Node	8
				Retroperitoneal Lymph Node	3
				Lung	1
				Adrenal	2

**Table 2:** Top 100 genes differentially expressed in metastatic samples compared to primary tumor samples

**Gene Symbol**	**Probe_ID**	**d_Value**	**Fold Change**
EIF1A	34278_at	19.46	3.56
AR	1577_at	16.04	10.09
AK3	48822_s_at	15.18	3.03
EIF1A	663_at	14.17	2.69
PABPC1	44806_at	14.17	3.57
---	45092_at	13.16	3.66
HSPD1	37720_at	12.80	2.80
---	54219_at	12.78	3.23
LCHN	58324_at	12.39	3.49
MLL5	58271_at	12.15	2.45
---	59350_at	11.98	3.22
---	49558_at	11.93	2.73
LARP	41829_at	11.60	3.14
IBTK	52482_at	11.41	3.30
GIT1	43805_f_at	11.41	2.49
---	56056_at	11.31	2.64
FLJ20736	64662_at	11.21	4.32
AR	1578_g_at	11.20	6.19
RALA	39253_s_at	11.19	3.61
HN1	56429_g_at	11.17	2.89
---	54236_at	11.04	3.82
HSPCA	32316_s_at	10.94	2.68
NUCKS	59778_f_at	10.93	2.44
SOD1	36620_at	10.87	2.16
K-ALPHA-1	32272_at	10.72	2.23
---	46558_at	10.70	4.70
---	33207_at	10.62	2.25
VPS28	43061_i_at	10.61	2.19
BASP1	32607_at	10.57	4.15
CBX4	51842_at	10.49	3.06
---	43680_at	10.45	4.85
GRB2	33855_at	10.44	2.55
---	63147_at	10.43	3.86
UGT2B15	63915_f_at	10.35	3.07
METTL2	48730_s_at	10.26	2.24
---	59101_at	10.11	7.12
MLL5	58690_at	10.02	2.24
G3BP	41133_at	10.00	2.25
AK3	32331_at	9.96	2.31
H2AV	39092_at	9.92	3.25
SDCCAG3	43014_at	9.91	6.04
FLJ10613	59989_s_at	9.82	2.20
YY1	891_at	9.70	2.08
---	49326_at	9.59	2.20
---	42646_at	9.50	5.45
LOC90462	54342_at	9.44	4.55
---	55393_at	9.42	3.51
DDX17	41260_at	9.35	4.97
---	63115_at	9.34	3.07
---	57160_at	9.34	2.11
ETR101	36097_at	-6.98	-3.85
MAGED2	34859_at	-7.00	-2.22
---	50411_at	-7.00	-3.57
MGC4342	48094_at	-7.01	-2.22
HT023	43461_g_at	-7.01	-2.78
SFRP1	32521_at	-7.02	-6.25
CRYL1	56407_at	-7.04	-2.04
REA	37364_at	-7.05	-2.17
ARL5	59499_at	-7.08	-2.50
NY-REN-45	57833_s_at	-7.09	-2.08
POMT1	46723_at	-7.12	-3.23
MRC2	63996_at	-7.13	-2.38
B3GALT3	53879_at	-7.16	-3.13
NR4A1	280_g_at	-7.21	-7.14
MUM2	65822_at	-7.23	-2.38
LOC113246	46712_at	-7.31	-2.17
GLTSCR2	61109_at	-7.33	-2.44
FLJ20542	50164_at	-7.36	-3.13
APCDD1	56272_at	-7.42	-2.70
AIM1	32112_s_at	-7.45	-3.70
FOS	1916_s_at	-7.51	-7.69
FOS	1915_s_at	-7.56	-9.09
PILB	43370_at	-7.56	-2.08
C20orf178	45298_at	-7.59	-2.13
B3GAT1	65859_at	-7.60	-2.13
FLJ10283	46261_at	-7.68	-2.27
RAB34	45269_at	-7.71	-10.00
FLJ20069	61701_at	-7.71	-3.23
PYGB	59669_at	-7.77	-2.70
RPS27L	56410_at	-7.80	-2.27
TOMM20	36198_at	-7.91	-2.13
---	43819_g_at	-7.91	-3.13
STAT6	41222_at	-7.92	-2.78
SELM	64449_at	-7.95	-9.09
BOC	52999_at	-8.15	-4.17
---	44746_at	-8.20	-2.13
SMBP	46307_at	-8.28	-3.23
WNT5B	61330_at	-8.34	-3.33
FLJ22386	50198_at	-8.43	-4.55
WNT5B	58787_at	-8.61	-2.27
ZDHHC4	45807_at	-8.81	-2.13
YF13H12	36170_at	-8.84	-2.08
HPIP	38063_at	-9.04	-2.33
ANXA11	55664_at	-9.16	-2.94
WNT5B	66142_s_at	-9.33	-4.17
WAS	38963_i_at	-9.43	-2.50
JFC1	44820_f_at	-9.45	-3.33
JFC1	48805_f_at	-9.48	-2.44
WNT5B	61292_s_at	-9.86	-7.14
CIRBP	39864_at	-10.94	-3.70

Gene expression data from the Affymetrix platform for 25 metastatic and 64 primary tumor samples was analyzed for differential gene expression by SAM. The differentially expressed genes with the lowest FDR were sorted by fold change. The top 100 genes, organized by fold change are shown.

**Table 3:** Transcripts with median values with at least 3 fold difference between metastatic and primary tumor samples

**Gene Symbol**	**Probe_ID**	**P1**	**P2**	**P3**	**P4**
**Upregulated Transcripts**					
HBB	32052_at	22.37	5.78	13.25	56.28
SPP1	34342_s_at	24.16	26.78	4.75	5.39
HBA1///HBA2	31525_s_at	15.14	4.47	13.65	108.11
LGR4	43585_at	7.39	7.43	20.89	24.82
AR	1577_at	14.35	12.97	12.24	14.78
PRO1073	49666_s_at	4.56	13.25	10.01	13.5
UTRN	42646_at	10.11	6.02	12.11	16.31
HNT	59070_at	5.37	9.69	12.08	13.67
SDCCAG3	43014_at	7.99	8.57	11.24	17.32
LOC64744	42739_at	7.3	9.64	9.57	14.51
---	1089_i_at	5.06	4.14	12.12	22.01
SPP1	2092_s_at	14.05	12.94	3.35	4.07
UBE2H	58777_at	9.5	7.45	6.55	15.13
SRPK1	63687_at	6.06	4.36	10.61	12.82
NCK2	33003_at	5	9.14	8.5	7.34
HIST1H3H	36757_at	7.26	17.07	8.47	5.61
PPP4R2	48663_at	5.09	6.97	8.59	16.16
C8orf16	47339_at	6.54	8.15	9.53	7.39
---	55943_at	3.41	7.47	15.31	8.01
---	64642_s_at	8.25	6.42	7.04	10.6
EP400	47518_at	5.94	9.27	4.51	9.32
GOLT1A	45144_at	3.9	6.17	8.37	12.32
---	52853_g_at	9.83	7.1	6.25	7.03
LOC284058	44791_at	8.25	10.17	4.55	5.86
DAPK1	51580_at	3.42	6.04	8.03	11.32
NFATC2IP	38864_at	3.26	4.83	9.58	9.19
SEL1L	40689_at	4.71	7.84	6.13	10.94
TM4SF9	47746_at	3.43	6.26	8.92	7.52
MLLT2	65205_at	3.43	7.13	6.57	13.01
SC4MOL	46802_at	22.91	7.35	5.62	6.17
---	62671_at	6.38	7.13	5.74	11.03
BIRC6	46558_at	5.67	8.59	7.5	5.92
MAP4K4	51474_at	4.86	4.32	8.7	8.52
MLLT2	53300_at	4.65	3.99	9.39	8.1
---	52851_at	8.71	5.94	6.35	6.25
MRRF	51635_at	4.23	4.87	7.39	8.23
ACAS2	62783_at	4.29	6.14	7.02	5.9
---	60658_at	3.4	6.84	5.19	9.1
SUMO1	49551_at	4.05	7.2	4.77	7.75
AR	1578_g_at	7.56	4.86	5.32	6.37
GALNT7	59101_at	8.41	4.12	5.11	6.54
GPR75	44203_at	5.14	8.32	3.9	6.31
TBL1XR1	65001_r_at	3.53	12.3	4.06	7.19
HSD17B12	43292_at	4.74	8.88	3.64	6.28
MRPS28	43095_at	5.79	5.39	5.58	5.14
FN1	64719_at	27.07	6.02	4.05	4.93
GPR158	44214_at	7.21	3.33	4.32	6.62
---	48069_at	6.27	9.88	3.38	4.55
FLJ21657	58778_at	4.34	5.6	6.17	5.18
MLL5	43301_at	4.76	3.61	5.87	10.34
---	55761_at	3.78	4.88	5.65	6.93
DLG1	47231_at	3.4	4.77	6.22	5.7
MYO5B	63281_r_at	3.29	6.17	4.29	6.84
---	49268_at	3.55	19.86	3.61	6.75
FUS	43501_at	3.93	3.78	6.42	8.97
CCDC35	54684_at	4.9	8.14	3.55	5.43
---	43435_at	6.85	4.83	4.82	5.49
SMA4	32921_at	4.68	5.53	5.74	4.26
NCOA1	45953_at	6.53	4.13	3.58	6.06
S100A8	41096_at	4.22	5.89	3.8	22.58
PRKCBP1	53493_at	4.65	7.37	4.5	5.35
RNPC2	65083_at	3.18	3.96	6.01	9.19
CAMSAP1	62630_at	4.45	5.8	3.36	5.36
EEF1G	41903_at	5.19	4.58	4.31	5.34
EIF5	51379_at	3.44	4.08	5.62	11.07
MAML3	49879_at	3.39	3.22	10.27	5.87
C21orf106	59651_at	3.19	4.02	5.23	6.44
VCIP135	42715_at	3.37	3.61	5.52	8.55
FOXO3A	55502_at	3.48	4.37	6.97	4.74
C7orf20	49143_s_at	4.23	4.62	4.41	5.78
GNMT	46482_at	3.59	4.84	4.24	4.64
DONSON	48549_at	4.1	3.58	4.66	5.28
---	43436_g_at	4.98	3.75	3.58	5.09
PKP4	66327_at	3.31	3.88	4.56	6.2
PCBP2	55393_at	3.73	3.19	4.36	6.29
CPEB4	57169_at	3.7	3.92	4.14	4.48
CUGBP1	34683_at	4.26	3.76	3.13	4.78
FALZ	47458_at	4.21	3.65	3.82	4.09
---	51586_at	3.51	4	4.99	3.89
RALA	39253_s_at	3.92	4.3	3.29	3.85
MLL5	45092_at	4.36	3.21	4.48	3.39
PABPC1	44806_at	3.74	3.98	4.2	3.07
EIF1AX	34278_at	3.99	3.47	3.84	3.19
C7orf2	42173_at	3.15	3.27	5.07	4.04
---	63147_at	3.25	5.4	3.12	4.04
RAD23B	41157_at	3.2	3.46	3.64	4.45
---	61037_at	3.44	3.56	3.47	3.73
NFATC1	39143_at	3.13	3.21	9.06	3.78
JARID1A	50532_at	3.22	3.32	3.54	4.12
PDLIM5	37366_at	3.02	3.58	3.42	3.16
					
**Downregulated Transcripts**					
NEFH	33767_at	-117.15	-147.36	-9.9	-17.18
C10orf116	32527_at	-35.49	-29.63	-46.85	-66.5
KLK11	40035_at	-23.65	-19.24	-39.73	-62.15
FAM3B	59657_at	-15.81	-27.92	-26.09	-25.97
PGM5	52140_at	-23.87	-26.5	-44.27	-17.72
MRGPRF	52946_at	-15.61	-18.57	-30.59	-70.95
KRT15	37582_at	-21.85	-20.74	-19.22	-33.68
PTN	34820_at	-11.62	-31.95	-10.24	-27.11
SELM	64449_at	-6.36	-8.4	-29.23	-39.36
MYLK	46276_at	-5.87	-15.22	-22.57	-20.86
SYNPO2	50361_at	-15.14	-15.77	-20.15	-84.14
KRT5	613_at	-13.21	-11.12	-22.66	-32.96
FOS	2094_s_at	-10.72	-25.75	-13.72	-16.45
PKP1	51214_at	-11.57	-16.34	-11.83	-17.85
---	42921_at	-9.96	-11.67	-15.61	-16.5
RAB34	45269_at	-14.36	-11.54	-17.49	-10.35
---	48927_at	-10.61	-14.93	-8.77	-21.91
ALOX15B	37430_at	-12.47	-12.41	-14.17	-9.1
FOS	1915_s_at	-7.59	-26.38	-11.03	-12.11
TMEM16G	62387_at	-9.63	-13.32	-12.59	-9.93
---	64676_at	-17.3	-9.39	-6.32	-13.05
SFRP1	32521_at	-13.1	-5.73	-8.29	-16.73
NDFIP2	60510_at	-7.2	-9.23	-11.72	-15.15
FHOD3	50298_at	-9.96	-12.84	-5.59	-10.96
WNT5B	61292_s_at	-8.72	-11.85	-5.42	-13.92
SYNPO2	48039_at	-11.04	-8.8	-12.64	-9.34
BOC	64423_s_at	-3.63	-8.16	-11.8	-54.66
SLC20A2	1137_at	-9.27	-5.08	-10.51	-12.61
COL8A2	52652_g_at	-7.95	-9.99	-11.56	-9.75
---	52678_at	-9.69	-9.99	-3.76	-17.93
FOS	1916_s_at	-7.58	-21.81	-6.93	-11.58
ARGBP2	51939_at	-7.77	-13.86	-10.4	-8.71
CTGF	64342_at	-4.21	-4.15	-20.44	-14.87
EPHB6	39930_at	-8.61	-9.66	-8.32	-19.41
SYNPO2	60532_at	-9.77	-5.54	-8.77	-9.03
NR4A1	280_g_at	-8.68	-13.49	-5.82	-8.58
DKFZP564O0823	54033_at	-4.67	-3.72	-11.83	-20
GSTO2	45609_at	-4.73	-6.81	-9.6	-16.18
---	49321_at	-7.91	-8.41	-9.24	-3.88
EGR3	40375_at	-9.89	-7.71	-8.49	-6.44
SYNPO2	61681_at	-7.85	-8.33	-4.56	-18.57
PI15	58361_at	-3.59	-4.26	-12.77	-11.74
FOSB	36669_at	-8.81	-6.27	-7.6	-8.39
OGN	43507_g_at	-3.56	-8.26	-7.19	-25.54
MOXD1	36834_at	-5.4	-11.7	-10	-3.85
LSAMP	43930_at	-3.05	-7.62	-9.76	-7.67
EGR2	37863_at	-7.7	-5.52	-7.23	-15.41
DKFZp686D0853	49770_at	-10.18	-7.66	-7.16	-4.39
LGP1	52826_at	-13.75	-5.94	-3.83	-8.11
ME3	35216_at	-7.45	-9.26	-6.54	-5.32
PPP1R14A	58774_at	-6.68	-6.14	-7.31	-7.87
FLJ22386	50198_at	-6.8	-3.64	-6.98	-6.65
NR4A1	279_at	-5.31	-8.04	-5.11	-8.48
WFDC1	64111_at	-3.79	-11.21	-6.64	-6.66
ZFP36	40448_at	-6.39	-6.86	-7.25	-3.61
CACHD1	43554_at	-6.68	-3.34	-17.46	-6.57
RLN1	35070_at	-6.78	-11.78	-5.14	-6.39
---	49975_at	-6.43	-6.16	-6.74	-10.11
CYBRD1	65852_at	-6.43	-4.79	-6.7	-7.23
PER3	53766_at	-15.43	-6.79	-5.56	-6.29
MN1	37283_at	-4.47	-7.36	-5.55	-7.48
DNCI2	35788_at	-4.2	-8.68	-3.02	-10.64
MRVI1	43966_at	-6.76	-5.28	-12.19	-6.09
AZGP1	35834_at	-6.32	-3.86	-38.18	-6.18
MGC14839	48949_at	-8.96	-4.19	-8.25	-3.61
SMTN	64499_s_at	-5.2	-15.22	-7.18	-4.42
HSPC157	50179_at	-5.66	-3.18	-6.63	-8.09
WFDC2	33933_at	-5.3	-6.5	-5.73	-6.81
BTG2	36634_at	-6.99	-3.13	-9.25	-5.22
AXIN2	64129_at	-4.97	-6.97	-7.18	-4.2
PDGFC	45217_at	-4.32	-7.53	-8.81	-3.97
MLLT10	63345_at	-7.2	-5.85	-5.9	-3.84
BMP7	49273_g_at	-4.58	-4.89	-6.82	-13.13
MCC	49504_r_at	-5.9	-5.71	-5.08	-5.84
HEXA	39340_at	-8.15	-5.65	-4.18	-5.88
GSTT2	1099_s_at	-6.47	-5.05	-6.8	-4.66
SSPN	65647_at	-5.4	-5.88	-3.12	-17.61
UPK3A	36379_at	-5.37	-4.71	-5.81	-6.91
PDE5A	54668_at	-4.44	-5.17	-5.87	-9.56
PSD3	63832_at	-3.19	-6.04	-4.98	-6.58
ALDH7A1	61965_at	-5.85	-5.14	-5.88	-3.13
FMOD	33431_at	-7.62	-4.3	-4.9	-6.04
TSPAN2	53693_at	-6.38	-4.49	-4.54	-6.77
DKFZP586H2123	40017_at	-6.52	-6.49	-4.32	-3.91
EFS	33883_at	-5.43	-3.58	-6.35	-5.18
PODN	63953_at	-4.16	-5.3	-4.84	-4.98
DUSP1	1005_at	-6.53	-16.66	-3.02	-3.19
SLC22A17	58898_s_at	-4.93	-5.81	-4.66	-4.44
CDH10	47535_at	-4.87	-3.19	-8.27	-4.65
---	64163_at	-3.66	-5.03	-4.79	-4.7
---	42587_at	-4.68	-4.62	-4.9	-3.45
TSPAN2	57331_at	-4.44	-8.06	-3.42	-4.71
SORBS1	56409_at	-5.45	-5.7	-3.17	-3.53
C21orf63	50658_s_at	-4.54	-3.36	-4.15	-5.31
NBL1	37005_at	-3.34	-4.27	-4.31	-6.36
CIRBP	39864_at	-4.38	-3.53	-4.19	-6.8
KLF4	48587_at	-3.77	-3.62	-4.57	-12.5
ZCSL2	45320_at	-3.1	-3.19	-5.88	-5.13
C12orf10	53911_at	-3.62	-4.44	-3.86	-6.46
CERKL	60314_at	-4.68	-3.03	-7.37	-3.62
NOV	39250_at	-3.2	-3.9	-4.38	-7.37
EPB41L5	60293_at	-4.33	-4.97	-3.06	-3.92
WNT5B	66142_s_at	-3.94	-3.87	-4.49	-4.16
ACYP2	64090_s_at	-3.36	-4.33	-3.68	-5.82
C9orf103	56186_at	-3.14	-4.62	-4.03	-3.73
FBXO2	57811_at	-3.51	-3.37	-4.16	-5.33
CD38	40323_at	-3.25	-3.37	-4.27	-4.27
BCAS1	37821_at	-4.96	-3.19	-4.26	-3.34
TMSL8	36491_at	-3.03	-4.11	-3.45	-7.67
ISL1	39990_at	-3.12	-3.78	-3.61	-3.91
HSPB8	56474_at	-3.45	-3.87	-3.04	-7.5
B3GALT3	53879_at	-3.04	-4.02	-3.77	-3.48
CYBRD1	50955_at	-3.7	-3.51	-3.21	-5.6
EFEMP2	63644_at	-3.25	-3.91	-3.28	-3.97
TU3A	45260_at	-3.14	-3.94	-3.22	-4.82
LOC57228	34176_at	-3.68	-5.3	-3.41	-3.16
IER2	36097_at	-4.79	-3.2	-3.11	-3.88
DKFZP564K1964	65860_at	-3.53	-3.11	-3.52	-4.62

A patient-specific median expression value was calculated from the multiple samples for each patient. These median values were then compared to the primary tumor expression value and those genes with 3-fold difference between metastatic and primary prostate cancer is shown.

**Table 4:** GO and pathway annotation of genes and pathways altered in metastatic prostate cancer

**Probe ID**	**Gene Symbol**	**Overall FC**	**FC_P1**	**FC_P2**	**FC_P3**	**FC_P4**
***Bone remodeling***						
34342_s_at	SPP1	24.7	24.16	26.78	4.75	5.39
2092_s_at	SPP1	11.8	14.05	12.94	3.35	4.07
47958_r_at	ANKH	-2	-2.23	-2.31	-2.03	-3.66
65035_at	TF1P11	-2.31	-2.28	-2.63	-2.97	-3.77
44596_at	TWIST2	-2.45	-2.7	-2.12	-2.94	-2.35
40333_at	BMP4	-2.5	-2.86	-2.89	-3	-5.23
49273_g_at	BMP7	-2.81	-4.58	-4.89	-6.82	-13.13
***Cell Adhesion***						
34342_s_at	SPP1	24.7	24.16	26.78	4.75	5.39
2092_s_at	SPP1	11.8	14.05	12.94	3.35	4.07
64719_at	FN1	7.5	27.07	6.02	4.05	4.93
59070_at	HNT	5	5.37	9.69	12.08	13.67
62628_at	PCDHGC3	3.4	2.24	5.44	2.97	2.42
44892_at	MLLT4	3.3	2.63	2.07	6.21	7.48
47064_at	HNT	3.27	2.98	4.37	8.07	8.4
66327_at	PKP4	2.79	3.31	3.88	4.56	6.2
35246_at	TYRO3	2.4	-5.43	-3.58	-6.35	-5.18
659_g_at	THBS2	1.9	2.39	2.37	2.26	2.7
59623_at	PCDH18	-2.22	-2.09	-2.89	-2.17	-2.39
53497_at	ITGB8	-2.38	-2.88	-2.69	-3.51	-13.81
60876_at	COL8A2	-2.38	-2.26	-3.26	-3.61	-2.25
47007_s_at	NINJ2	-2.63	-2.55	-3.4	-3.3	-5.93
103_at	THBS4	-2.7	-2.02	-3.37	-2.16	-3.26
46520_at	ROBO2	-2.86	-2.58	-4.96	-4.14	-4.46
45939_at	CNTN3	-3.33	-3.38	-2.14	-7.41	-11.94
47535_at	CDH10	-3.7	-4.87	-3.19	-8.27	-4.65
56192_at	PCDH7	-3.7	-2.5	-5.33	-5.74	-3
52999_at	BOC	-4.17	-2.42	-8.8	-12.25	-6.16
43930_at	LSAMP	-4.35	-3.05	-7.62	-9.76	-7.67
33883_at	EFS	-4.76	-5.43	-3.58	-6.35	-5.18
64342_at	CTGF	-5.26	-4.21	-4.15	-20.44	-14.87
64423_s_at	BOC	-6.67	-3.63	-8.16	-11.8	-54.66
52652_g_at	COL8A2	-10	-7.95	-9.99	-11.56	-9.75
51214_at	PKP1	-14.29	-11.57	-16.34	-11.83	-17.85
52140_at	PGM5	-25	-23.87	-26.5	-44.27	-17.72
***Cell_cycle***						
47231_at	DLG1	3.57	3.4	4.77	6.22	5.7
45574_g_at	TPX2	3.49	11.09	2.57	3.24	8.6
54219_at	7-Sep	3.23	3.3	2.79	2.17	3.27
53998_at	CLASP2	3.09	2.67	2.84	4.18	4.13
37933_at	RBBP6	3.08	2.27	5.42	7.52	8.79
53568_at	7-Sep	2.97	3.94	2.4	3.53	2.49
51815_at	TERF1	2.54	2.11	4.78	5.1	3.51
1797_at	CDKN2D	2.41	3.65	2.76	2.46	2.47
50084_at	DNCH1	2.29	2.42	2.15	2.11	2.29
66955_at	EML4	2.13	2.22	3.3	6.8	5.09
1833_at	CDK2	2.12	3.14	2.56	2.05	2.45
52744_at	HRPT2	2.07	2.73	2.62	3.4	3.36
60568_at	BCL2	2.07	2.18	3.27	3.35	3.75
41632_at	E2F3	2.05	2.34	2.04	2.2	2.15
59821_at	BCL2	1.87	2.89	3.13	3.58	3.86
36101_s_at	VEGF	1.53	2.5	2.38	2.53	3.06
63158_at	GRLF1	1.27	3.55	2.63	2.3	3.45
65908_at	CHES1	-2.1	-3.3	-2.11	-2.34	-5.51
46664_at	PYCARD	-2.14	-2.36	-2.06	-2.94	-2.08
48980_at	ZAK	-2.25	-2.02	-4.8	-2.06	-2.07
36838_at	KLK10	-2.38	-3.38	-2.94	-2.07	-6.13
50199_s_at	RGC32	-2.73	-2.49	-3.64	-5.45	-4.17
39780_at	PPP3CB	-2.74	-3.82	-2.36	-3.06	-2.85
33864_at	ZMYND11	-2.78	-4.77	-2.85	-3.79	-3.79
49504_r_at	MCC	-3.45	-5.9	-5.71	-5.08	-5.84
37005_at	NBL1	-3.83	-3.34	-4.27	-4.31	-6.36
234_s_at	PTN	-3.86	-2.92	-5.02	-2.15	-3.33
37283_at	MN1	-4.63	-4.47	-7.36	-5.55	-7.48
45217_at	PDGFC	-4.82	-4.32	-7.53	-8.81	-3.97
1005_at	DUSP1	-6.16	-6.53	-16.66	-3.02	-3.19
36669_at	FOSB	-7.85	-8.81	-6.27	-7.6	-8.39
34820_at	PTN	-12.55	-11.62	-31.95	-10.24	-27.11
37430_at	ALOX15B	-21	-12.47	-12.41	-14.17	-9.1
***Transcription***						
1577_at	AR	10	14.35	12.97	12.24	14.78
65001_r_at	TBL1XR1	7	3.53	12.3	4.06	7.19
1578_g_at	AR	6.18	7.56	4.86	5.32	6.37
42733_i_at	FOXP1	5.6	2.61	5.92	14.54	14.09
52769_at	POLR2A	5.1	2.93	4.04	12.61	11.14
54342_at	ZNF605	4.55	2.75	6.89	4.85	4.04
65083_at	RNPC2	4.1	3.18	3.96	6.01	9.19
42734_r_at	FOXP1	3.5	2.01	4.49	7.53	8.6
48423_at	ZNF621	3.4	2.11	8.51	3.74	7.33
51543_at	ZNF395	3.32	2.99	5.52	6.73	6.51
44546_at	ZNF148	3	3.32	2.59	4.72	4.18
49633_at	HES6	3	5.76	2.26	2.34	6.72
51842_at	CBX4	3	3.32	2.61	5.21	3.08
54981_r_at	SFPQ	3	2.27	4.87	4.9	5.33
60076_at	SOX4	3	3.32	3.77	2.09	2.72
43580_at	MORF4L2	2.93	2.42	3.49	3.38	6.52
34715_at	FOXM1	2.84	6.82	2.17	2.9	2.99
40674_s_at	HOXC6	2.81	3.09	2.48	6.67	4.56
56981_at	ZKSCAN1	2.77	2.65	2.4	2.6	4.04
55293_at	ADNP	2.73	2.44	3.6	3.98	3.9
43545_at	ZNF281	2.7	2.17	3.21	3.05	4.19
32653_at	BRD8	2.56	2.11	3.73	3.22	4.45
53846_at	FLJ21616	2.55	3.1	2.96	5.41	5.92
45953_at	NCOA1	2.42	6.53	4.13	3.58	6.06
46006_at	ERCC8	2.35	2.35	2.7	3.36	5.84
50911_at	RLF	2.35	2.18	2.38	4.07	4.92
58641_at	MAML3	2.33	2.86	2.31	5.61	5.44
42571_at	MORF4L2	2.32	2.21	3.7	3.99	3.48
43120_at	MLL3	2.32	2.55	2.48	2.14	2.74
31437_r_at	ESR2	2.3	2.06	3.06	3.13	4.09
50532_at	JARID1A	2.3	3.22	3.32	3.54	4.12
44939_at	MLL3	2.22	2.55	2.48	2.14	2.74
55502_at	FOXO3A	2.21	2.44	3.6	3.98	3.9
52328_at	SP3	2.2	2.13	2.62	3.38	3.11
54220_r_at	NLK	2.16	2.39	4.23	3.94	4.41
41632_at	E2F3	2.04	2.34	2.04	2.2	2.15
66313_at	HIPK1	2	2.09	2.85	2.95	3.98
42193_r_at	---	1.9	2.16	2.04	2.66	3.29
39540_at	ZBTB7A	1.89	2.42	3.27	3.03	2.61
63158_at	GRLF1	1.79	3.55	2.63	2.3	3.45
65908_at	CHES1	-2.13	-3.3	-2.11	-2.34	-5.51
36770_at	STAT2	-2.17	-2.3	-3.47	-2.82	-3.75
64963_at	EYA4	-2.17	-3.06	-3.31	-2.55	-5.01
65945_at	PNRC2	-2.17	-2.08	-2.26	-2.61	-2.24
1454_at	SMAD3	-2.27	-2.17	-2.51	-2.02	-2.07
54658_at	PSPC1	-2.33	-3.18	-2.22	-3.63	-13.16
44596_at	TWIST2	-2.5	-2.7	-2.12	-2.94	-2.35
46704_at	KLF3	-2.63	-2.38	-2.29	-2.19	-3.33
51253_at	ZBTB4	-2.63	-2.92	-3.18	-2.63	-3.93
287_at	ATF3	-2.78	-2.11	-2.33	-2.63	-3.3
33864_at	ZMYND11	-2.78	-4.77	-2.85	-3.79	-3.79
63526_f_at	KLF6	-2.78	-3.21	-2.48	-2.71	-3.33
64932_at	VPS36	-2.86	-4.56	-2.88	-2.48	-2.27
45680_at	ZNF537	-2.94	-2.77	-3.37	-2.78	-3.74
43431_at	SOX2	-3.03	-2.09	-3.8	-7.43	-7.34
47620_at	XAB2	-3.33	-2.7	-8.25	-7.6	-8.49
465_at	HTATIP	-3.45	-2.52	-2.37	-4.49	-2.47
37863_at	EGR2	-4.55	-7.7	-5.52	-7.23	-15.41
48587_at	KLF4	-4.76	-3.77	-3.62	-4.57	-12.5
36634_at	BTG2	-5.26	-6.99	-3.13	-9.25	-5.22
279_at	NR4A1	-6.25	-5.31	-8.04	-5.11	-8.48
280_g_at	NR4A1	-7.14	-8.68	-13.49	-5.82	-8.58
40375_at	EGR3	-8.33	-9.89	-7.71	-8.49	-6.44
53766_at	PER3	-9.09	-15.43	-6.79	-5.56	-6.29
***MAP Kinase Pathway***						
1562_g_at	DUSP8	5.8	2.42	5.01	16.6	5.86
1104_s_at	HSPA1A/HSPA1B	4.1	7.81	2.77	2.81	5.02
51474_at	MAP4K4	3.57	4.86	4.32	8.7	8.52
60658_at	---	3.5	-3	-2.96	-3.6	-3.73
50375_at	SOS1	3	2.97	4.08	4.57	3.79
33855_at	GRB2	2.55	2.29	2.61	3.85	2.1
42838_f_at	MAP3K8	2.3	2.25	3.4	2.78	2.08
54220_r_at	NLK	2.16	2.39	4.23	3.94	4.41
790_at	NGFB	1.86	2.08	2.35	3.37	2.9
48980_at	ZAK	-2.27	-2.02	-4.8	-2.06	-2.07
43053_g_at	PAK1	-2.7	-6.31	-2.46	-2.83	-2.11
39780_at	PPP3CB	-2.78	-3.82	-2.36	-3.06	-2.85
468_at	FGF13	-2.78	-4.35	-4.35	-2.11	-2.56
65904_at	---	-2.94	-2.05	-3.48	-4.18	-3.43
1292_at	DUSP2	-3.13	-3.72	-3.66	-3.17	-2.19
57299_s_at	RRAS	-3.23	-3	-2.96	-3.6	-3.73
1970_s_at	FGFR2	-5.56	-2.55	-13.13	-8.88	-4.92
1005_at	DUSP1	-6.25	-6.53	-16.66	-3.02	-3.19
279_at	NR4A1	-6.25	-5.31	-8.04	-5.11	-8.48
280_g_at	NR4A1	-7.14	-8.68	-13.49	-5.82	-8.58
1916_s_at	FOS	-7.69	-7.58	-21.81	-6.93	-11.58
1915_s_at	FOS	-9.09	-7.59	-26.38	-11.03	-12.11
2094_s_at	FOS	-16.67	-10.72	-25.75	-13.72	-16.45

Genes whose median value shows at least 2 fold change in every metastatic patient were annotated by NIH's DAVID tool. Genes belonging to selected GO categories with a significant number of differentially expressed genes are shown. *Overall_FC*, the fold change in the mean expression value of all metastatic samples compared to the primary samples;*FC_P1, FC_P2, FC_P3, FC_P4*; a patient-specific median expression value was calculated using all the samples from each patient. This was compared to the median value for the primary tumors. Patient P4 had only sample and this was considered the median.
